# Heroin versus cocaine: opposite choice as a function of context but not of drug history in the rat

**DOI:** 10.1007/s00213-018-5115-1

**Published:** 2018-11-15

**Authors:** Maria Teresa De Luca, Christian Montanari, Maria Meringolo, Laura Contu, Michele Celentano, Aldo Badiani

**Affiliations:** 1 Department of Physiology and Pharmacology Vittorio Erspamer, Sapienza University of Rome, Rome, Italy; 2 Sussex Addiction Research and Intervention Centre (SARIC), School of Psychology, University of Sussex, Sussex, UK; 3 Sussex Neuroscience, University of Sussex, Sussex, UK

**Keywords:** Drug addiction, Drug abuse, Drug dependence, Environment, Context, Self-administration, Drug choice, Psychostimulants, Opiates, Opioids

## Abstract

**Rationale:**

Previous studies have shown that rats trained to self-administer heroin and cocaine exhibit opposite preferences, as a function of setting, when tested in a choice paradigm. Rats tested at home prefer heroin to cocaine, whereas rats tested outside the home prefer cocaine to heroin. Here, we investigated whether drug history would influence subsequent drug preference in distinct settings. Based on a theoretical model of drug-setting interaction, we predicted that regardless of drug history rats would prefer heroin at home and cocaine outside the home.

**Methods:**

Rats with double-lumen catheters were first trained to self-administer either heroin (25 μg/kg) or cocaine (400 μg/kg) for 12 consecutive sessions. Twenty-six rats were housed in the self-administration chambers (thus, they were tested at home), whereas 30 rats lived in distinct home cages and were transferred to self-administration chambers only for the self-administration session (thus, they were tested outside the home). The rats were then allowed to choose repeatedly between heroin and cocaine within the same session for seven sessions.

**Results:**

Regardless of the training drug, the rats tested outside the home preferred cocaine to heroin, whereas the rats tested at home preferred heroin to cocaine. There was no correlation between drug preference and drug intake during the training phase.

**Conclusion:**

Drug preferences were powerfully influenced by the setting but, quite surprisingly, not by drug history. This suggests that, under certain conditions, associative learning processes and drug-induced neuroplastic adaptations play a minor role in shaping individual preferences for one drug or the other.

## Introduction

Drug use is rarely limited to a single substance, polydrug use being more of a rule than an exception (e.g., Sample 1977; Brecht et al. 2008; Badiani et al. 2015; John et al. 2018). In particular, it is well known that most individuals with heroin use disorder also use cocaine and vice versa (Leri et al. 2003, 2005; Kosten et al. 1986; Levin et al. 1996). Yet, polydrug use does not preclude the forming of preferences for one drug or another (Harford 1978; Gossop and Connell 1975). The mechanisms responsible for these preferences are still poorly understood, leaving aside obvious constraints, such as those deriving from law, market availability, and street price (e.g., Jofre-Bonet and Petry 2008). However, we have shown that the context of use (the setting) plays an important role in modulating drug preference in an animal model of drug self-administration (Caprioli et al. 2009). In this experiment, we trained two groups of rats to self-administer heroin and cocaine on alternate days and then we gave them the opportunity to choose repeatedly between the two drugs within the same session. One of the two groups self-administered the drugs at home (that is, these rats resided in the self-administration chambers). The other group was transferred to the self-administration chambers only for the test sessions (thus, these rats self-administered the drug outside the home). Despite the fact that the self-administration environment was physically identical for the two groups, we found striking differences in their drug preferences. Most rats tested at home preferred heroin to cocaine. In contrast, most rats tested outside the home preferred cocaine to heroin. There are at least three possible explanations for this phenomenon. The first one relates to basic associative learning processes that have been shown to play an important role in drug craving (Childress et al. 1993; Grimm et al. 2001). Since heroin and cocaine were paired with different levers (and their respective light cues) during training, the rats might have developed a setting-dependent bias for one of the two levers. This bias might have expressed itself during the choice phase. A second possibility relates to the fact that during training the rats took different amounts of cocaine versus heroin, as function of setting (see also Caprioli et al. 2007, Caprioli et al. 2008). This might have induced distinct neuroadaptations in the two settings, as it has been shown that repeated exposure to opiates such as morphine produces neuroplastic changes opposite to those produced by psychostimulants such as cocaine (Robinson and Kolb 2004; Becker et al. 2017). Opposite neuroplastic adaptations might have resulted, during the choice phase, in greater craving for heroin at home and for cocaine outside the home. Finally, it is possible that drug preferences were the result of a more fundamental interaction between drug and setting. We have in fact proposed (Badiani 2013) that the rewarding effect of any drug is decreased in the presence of a “mismatch” between the interoceptive information produced by central and peripheral drug effects and the exteroceptive information (the setting). In the case of cocaine, for example, a mismatch would occur when this drug, which produces arousal and sympathetic activation (Billman 1995; Sofuoglu and Sewell 2009), is taken in a quiet domestic environment. The opposite would occur when heroin, which produces sedation and parasympathomimetic effects (Haddad and Lasala 1987; Thornhill et al. 1989), is taken outside the home, that is in exciting, potentially dangerous contexts.

The aim of the present study was to investigate the influence of setting on heroin versus cocaine choice as a function of drug history, using an unbiased procedure. Thus, we modified the procedure used by Caprioli et al. (2009) rats in two ways: (1) we tested independent groups of rats with a history of either heroin or cocaine self-administration; (2) during training, both levers were paired with the same drug so that no lever bias could develop before the choice phase. The three hypotheses highlighted above predict different results. If drug preferences were the result of associative learning processes or of neuroplastic changes relating to drug history, heroin-trained rats should prefer heroin to cocaine, regardless of setting, whereas cocaine-trained rats should prefer cocaine to heroin. Based on our working hypothesis, we predicted instead that rats would prefer heroin to cocaine at home, and cocaine to heroin outside the home, regardless of drug history.

## Methods and materials

### Animals

Seventy-two male Sprague-Dawley rats (Harlan, Italy), weighing 250–275 g at their arrival, were used in this study. Eleven rats were excluded because of catheter clogging (i.e., they failed the catheter patency test, see below) or sickness. Five rats that did not reach the criterion for the acquisition of self-administration (see “Data analysis and statistics”) were also excluded. Thus, only the data from 56 rats were included in the final analysis (N’s refer to these rats). Final sample size was determined based on a previous report with similar experimental design (Caprioli et al. 2009).

The rats were housed and tested in the same dedicated temperature- and humidity-controlled rooms, with free access (except during the test sessions) to food and water under a 14-h dark/10-h light cycle (lights off at 7:00 AM). After their arrival, the rats were housed two per cage for 7–10 days before the surgery. After the surgery, the rats were housed individually (see “General self-administration procedures”). The procedures were approved by the Italian Ministry of Health and were conducted in accordance with the European Community Directives (2010/63/EU), with the Italian Law on Animal Research (Decreto Legislativo 26/2014), and with the guidelines for the care and use of laboratory animals issued by the Italian Ministry of Health.

### Self-administration apparatus

The apparatus consisted of self-administration chambers (28.5-cm length, 27-cm width, and 32-cm height) made of transparent plastic (front and rear walls), aluminum (side-walls and ceiling), and stainless steel (grid floor). Plastic trays covered with pine wood shavings were placed under the cage floors. Each chamber was equipped with two retractable levers, positioned on the left hand wall 12.5 cm apart and 9 cm above the floor, two sets of three cue lights (red, yellow, and green), positioned above each lever, and a counterbalanced arm holding a double channel liquid swivels. Chambers and accessories were purchased from ESATEL S.r.l. (Rome, Italy). The self-administration chambers were placed within sound- and light-attenuating cubicles. Each chamber was connected via an electronic interface (ESATEL) to a syringe pump (Razel Scientific Instruments, St Albans, VT, USA) and to a Programmable Logic Controller (PLC; Allen Bradley, Milwaukee, WI, USA). Finally, the PLCs were connected to PCs running control software developed by Aries Sistemi S.r.l. (Rome, Italy).

### Surgery

The intravenous double-lumen catheters consisted of two 10.5 cm of silicone tubing (0.37-mm inner diameter, 0.94-mm outer diameter) sheathed, at 3.4 cm from its proximal end, by a 5-mm length of heat-shrink tubing. On the day of surgery, the rats received an intraperitoneal (i.p.) injection of 2.33 mg of xylazine hydrochloride (Rompun®, Bayer HealthCare) and an intramuscular injection of 14,000 IU of benzylpenicillin (Fournier Pharma, S. Palomba, Italy). The rats were then anesthetized with an i.p. injection of 0.56 ml/kg of Zoletil 100® (Virbac, Carros, France), containing tiletamine (50 mg/ml) and zolazepam (50 mg/ml). Using standard surgical procedures, the double-lumen catheters were inserted into the right jugular vein, so as to reach the right atrium with its proximal end, and was then secured to the surrounding soft tissues with silk thread. The distal end of the double-lumen catheters was passed subcutaneously in front of the left shoulder, externalized through a small incision at the nape of the neck, and connected to two L-shaped 22-gauge cannulas. The cannula was then secured to the rat’s skull using dental cement and stainless steel screws. After surgery, the rats were given 15 mg i.v. enrofloxacin (Baytril®, KVP Pharma + Veterinär produkte Gmbh, Kiel, Germany) in a double i.v. bolus. Catheters were flushed daily (at 1800 h) with 0.1 ml of a sterile saline solution containing 0.4 mg of enrofloxacin and 25 IU heparin (Marvecs Services, Agrate Brianza, Italy). At the end of the experiment, all rats underwent a catheter patency test in which they received two i.v. boluses of 40 mg/kg of thiopental sodium (Pharmacia Italia, Milan, Italy), one in each catheter lumens, with a 15-min interval between the two.

### Setting of self-administration

**Fig. 1:**
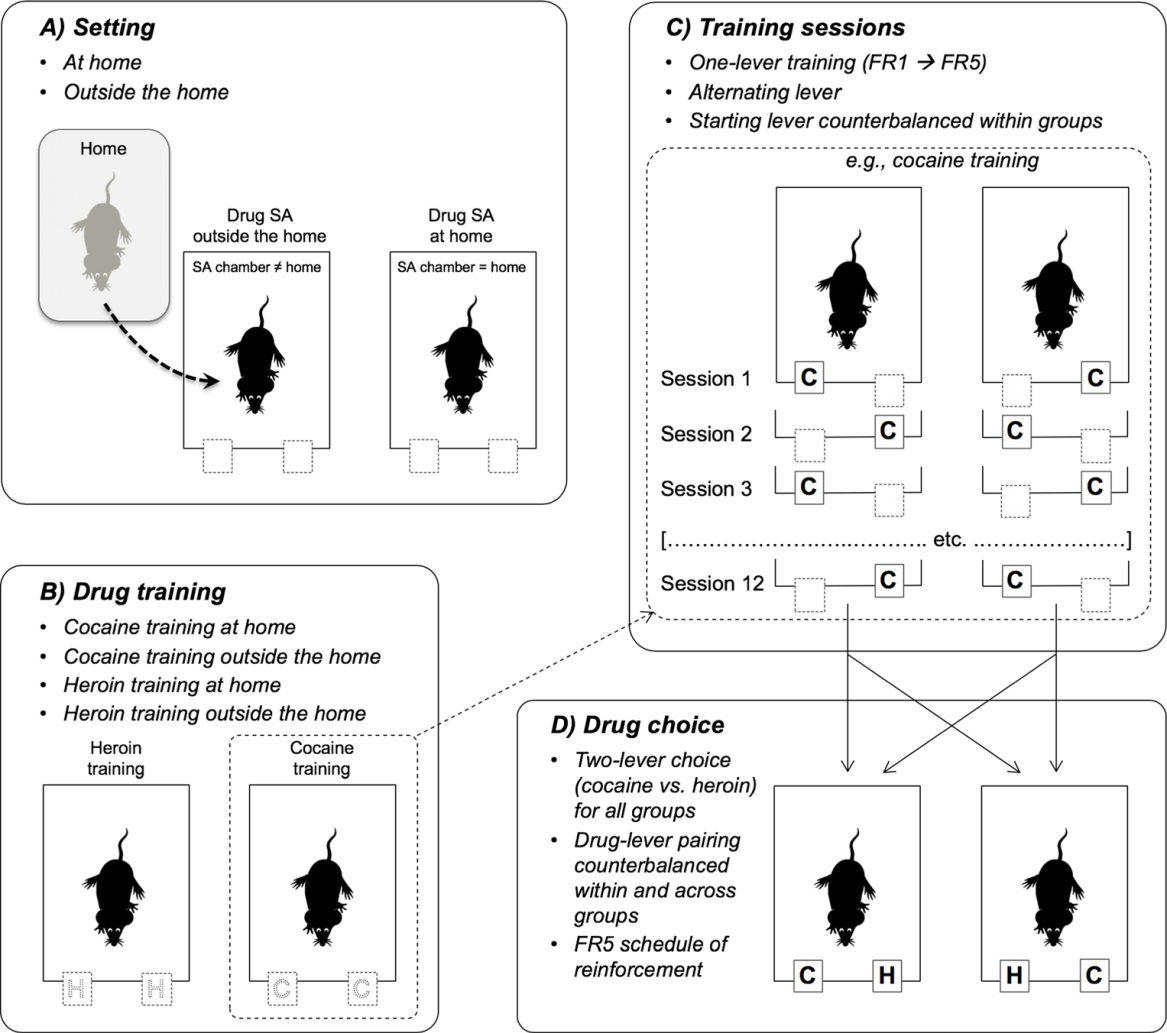
Schematic representation of the testing procedures. **a** Setting. **b** Self-administration training with either heroin or cocaine. **c** One-lever training sessions with alternating right and left lever. The inset details the counterbalanced sequence for cocaine-trained rats. The same was done for heroin-trained rats. **d** To ensure that the choice procedure was completely unbiased, the levers were also counterbalanced with respect to the last training session (see panel **c**). Thus, for some of the rats that had ended the training (session 12) with the right lever, the choice was between cocaine on the right and heroin on the left, whereas for the other rats, cocaine was on the left and heroin on the right. The same was done for the rats that had ended the training (session 12) with the left lever

The rats were allowed at least 7 days to recover from the surgery and were then randomly assigned to one of two groups, to be tested at home and outside the home, respectively. Home rats (*N* = 26) were individually housed in the self-administration chambers where they remained for the entire duration of the experiment. The other rats (*N* = 30) were individually housed in standard transparent plastic cages (40-cm length, 24.5-cm width, and 18-cm height; with stainless steel tops and flat bottoms covered with ground corncob bedding) and were transferred to the self-administration chambers immediately before the start of each testing session. *Thus, although the testing environment was physically identical for the two groups, it represented home for one group and a distinct environment for the other group* (see Fig. 1a).

### Procedures

#### General self-administration procedures

The self-administration sessions took place during the dark phase, between 1230 and 1630 h. The rats tested at home were connected to the infusion lines 3 h before the start of each session. The rats tested outside the home were transferred to the self-administration chambers immediately before the start of each session and their catheters were connected to the infusion lines. In both groups, the infusion pumps were activated during the 60 s preceding the start of each session, so as to fill the *dead volume* of the catheters’ lumina with the appropriate drug solutions (thus, no drug entered the rats’ bloodstream before the beginning of the session). During the training phase (see below), only one lumen was used in any given session, whereas during the choice phase (see below), both lumina were filled (one with heroin and the other with cocaine). At this time, food and water were removed from the chambers of rats tested at home, so that both groups were tested in the absence of food or water. All other husbandry and testing routines were identical for the two groups. Notice that throughout the experiments, the rats were *individually* housed and tested in the same dedicated testing room (thus, was no transport from one room to another and no disruption of social context or circadian rhythmicity) with ad libitum access to food and water (except during the test sessions).

The experiment included three phases: training (12 days), withdrawal (3 days), and choice (7 days).

#### Training phase (days 1–12)

The rats were randomly assigned to self-administer either heroin (*N* = 12 at home and *N* = 14 outside the home) or cocaine (*N* = 14 at home and *N* = 16 outside the home) for 12 consecutive daily 3-h sessions (see Fig. 1b, c). Unit doses of 25 μg/kg for heroin (Sigma, St. Louis, MO, USA) and 400 μg/kg for cocaine (Division of Neuroscience & Behavioral Research, NIDA, Bethesda, MD, USA) were dissolved in sterile saline to an infusion volume of 40 μl. These doses were selected on the basis of previous experiments (Caprioli et al. 2007a, 2008, 2009).

**Fig. 2:**
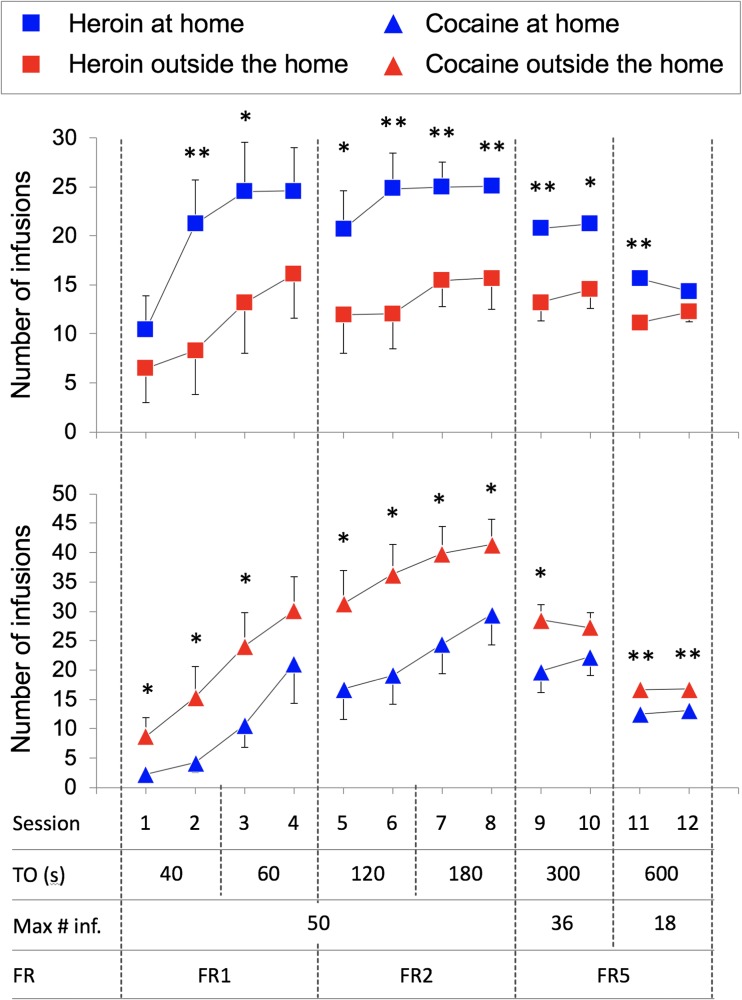
Mean (±SEM) number of infusions during the training phase for the heroin- and cocaine-trained groups, as a function of setting, time-out (TO) period, maximum number of infusions, and fixed ratio (FR). Single and double asterisks indicate significant effect setting (*p* < 0.05 and *p* < 0.01, respectively). See text for more detail about procedures and statistical analysis

At the start of each training session, *only one lever was extended* and the respective cue lights were turned on. The fixed ratio (FR), that is, the number of consecutive lever presses required to obtain a single infusion, increased during training from 1 (FR1) in sessions 1–4, to FR2 in sessions 5–8, and then to FR5 in sessions 9–12 (see Fig. 2). The 40 μl of drug solution was infused at a rate of 10 μl/s. After each infusion, the cue lights turned off and the lever retracted. The cue lights turned on and the lever extended again after a time-out (*T*) period. In order to achieve, by the end of the training phase, the schedule of reinforcement to be used during the choice sessions (see next subsection), the duration of the TO period was progressively increased over test sessions: 40 s in sessions 1–2, 60 s in sessions 3–4, 120 s in sessions 5–6, 180 s in sessions 7–8, 300 s in sessions 9–10, and 600 s in sessions 11–12. A synopsis of the schedule of self-administration during training is illustrated in Fig. 2.

It is important to stress that in each group, *both levers were alternatively paired with the drug and the starting lever was counterbalanced across rats*. That is, some rats started with the right lever on session 1 and ended with the left lever on session 12. The opposite occurred for the other rats (see Fig. 1c). Furthermore, the two lumina of the catheter were used alternatively, in a counterbalanced manner across sessions, to deliver the drug solution.

At the end of each session, food and water were given back to the rats at home and the rats in the outside-the-home condition were returned to their home cages.

#### Withdrawal phase (days 12–15)

After the end of the training phase, the rats were withdrawn from self-administration for 3 days (from the afternoon of day 12 to the morning of day 15). During this period, the rats were left undisturbed in their respective housing to ensure complete cocaine and heroin clearance (see Caprioli et al. 2009).

#### Choice phase (days 15–21)

After the withdrawal period, the rats underwent a choice phase consisting of seven sessions (3 h each) during which the rats were allowed to choose repeatedly between heroin (25 μg/kg) and cocaine (400 μg/kg) within session (see Fig. 1d). Notice that *the choice phase was the first time cocaine-trained rats had access to heroin and heroin-trained rats had access to cocaine*.

At the beginning of each choice session, both levers were extended and the respective cue lights turned on. One lever was paired with heroin and the other lever with cocaine, in a counterbalanced manner across rats. As during the last training sessions, the choice sessions were conducted on an FR5 schedule of reinforcement but in this case pressing on the cocaine lever reset the counter of the heroin lever and vice versa. Once the animal completed the FR5 task on one of the levers, the drug was administered, the respective cue lights turned off, and both levers retracted for a 600-s TO period, after which the levers were extended again and the lights turned on for the next trial. Thus, the rats could self-administer a maximum of 18 infusions per session.

To ensure that the choice procedure was completely unbiased, the levers were also counterbalanced with respect to the last training session (see Fig. 1c, d). Thus, for some of the rats that had ended the training (session 12) with the right lever, the choice was between cocaine on the right and heroin on the left, whereas for the other rats, cocaine was on the left and heroin on the right. The same was done for the rats that had ended the training (session 12) with the left lever. Furthermore, the infusion lines were counterbalanced across rats and sessions; that is, on the first choice sessions, some rats received cocaine through the anterior lumen and heroin through the posterior lumen, the opposite occurred on the following session, and so on; the reverse sequence was used for the other rats.

#### Catheter patency test

At the end of the experiment, all rats underwent a catheter patency test in which they received two i.v. boluses of 40 mg/kg of thiopental sodium (Pharmacia Italia, Milan, Italy), one in each catheter lumens, with a 15-min interval between the two. Five rats that failed the test (that is, that did not became ataxic within 5 s after thiopental) were excluded from the analyses.

### Data analysis and statistics

#### Training data

Five rats did not reach the criterion for self-administration (an average of at least three infusions of cocaine or heroin per session on the last four sessions on FR5) and were excluded from the analysis. Self-administration data for the training phase were analyzed using a three-way ANOVA for the between-subject factors *setting* (two levels: home vs. outside the home) and *drug* (two levels: cocaine vs. heroin) and the repeated-measure factor *training session* (12 levels). The effect of *setting* on heroin or cocaine self-administration for each session was estimated using one-tailed Student’s *t* tests (as the direction of the difference was predicted by the working hypothesis). The effect size was estimated by calculating partial eta-squared (*η*^2^), with critical values: 0.01–0.059 = small effect size, 0.06–0.13 = medium effect size, and > 0.14 = large effect size (Cohen 1988).

#### Choice data

The total number of choices was analyzed using a three-way ANOVA for the between-subject factors *setting* (two levels: home vs. outside the home) and *drug history* (two levels: cocaine and heroin) and the repeated-measure factor *choice session* (seven levels). The number of cocaine versus heroin choices could not be compared directly because the two variables were inversely related. Thus, we first calculated, for each rat, the ratio between the number of cocaine infusions and the total number of infusions (cocaine choice ratio) for the seven choice sessions. After arcsine transformation, these data were analyzed using a three-way ANOVA for the between-subject factors *setting* (two levels: home vs. outside the home) and *drug history* (two levels: cocaine and heroin) and the repeated-measure factor *choice session* (seven levels). Effect size was estimated by calculating *η*^2^. Furthermore, based on the proportion of cocaine infusions on the last three choice sessions, the rats were individually classified, using a straightforward bootstrapping procedure (Wilson 1927; Newcombe 1988), as cocaine-preferring, heroin-preferring, or non-preferring (*p*’s < 0.05).

The relationships between drug intake during training and drug preferences were assessed using linear correlation analysis.

## Results

### Drug self-administration training

Figure 2 illustrates the number of infusions during training for the heroin (left panel) and cocaine (right panel) groups, as a function of the setting. The ANOVA showed a significant *drug* × *setting* interaction (*F*_1, 52_ = 12.074, *p* < 0.001, *η*^2^ = 0.188) but no main effect of *drug* (*F*_1, 52_ = 3.263, *p* = 0.077, *η*^2^ = 0.059) or *setting* (*F*_1, 52_ = 0.131, *p* = 0.719, *η*^2^ = 0.003). There was also a significant *training session* × *drug* × *setting* interaction (*F*_11, 52_ = 2.044, *p* = 0.023, *η*^2^ = 0.038). Follow-up analysis showed a significant effect of *setting* for both heroin (*F*_1, 24_ = 6.954, *p* = 0.014, *η*^2^ = 0.225) and cocaine (*F*_1, 28_ = 6.101, p = 0.02, *η*^2^ = 0.179) groups, indicating that, consistent with previous findings (and despite the constraints in the maximum number of infusions), rats at home self-administered more heroin than rats outside the home whereas rats outside the home self-administered more cocaine than rats at home. The results of pair-wise comparisons for the effect of *setting* in each session are illustrated in Fig. 2. Notice that the narrowing of group differences in the last training sessions was the result of a ceiling effect due to the constraints in the maximum number of infusions (see “Methods and materials” section and Fig. 2).

### Drug choice

**Fig. 3:**
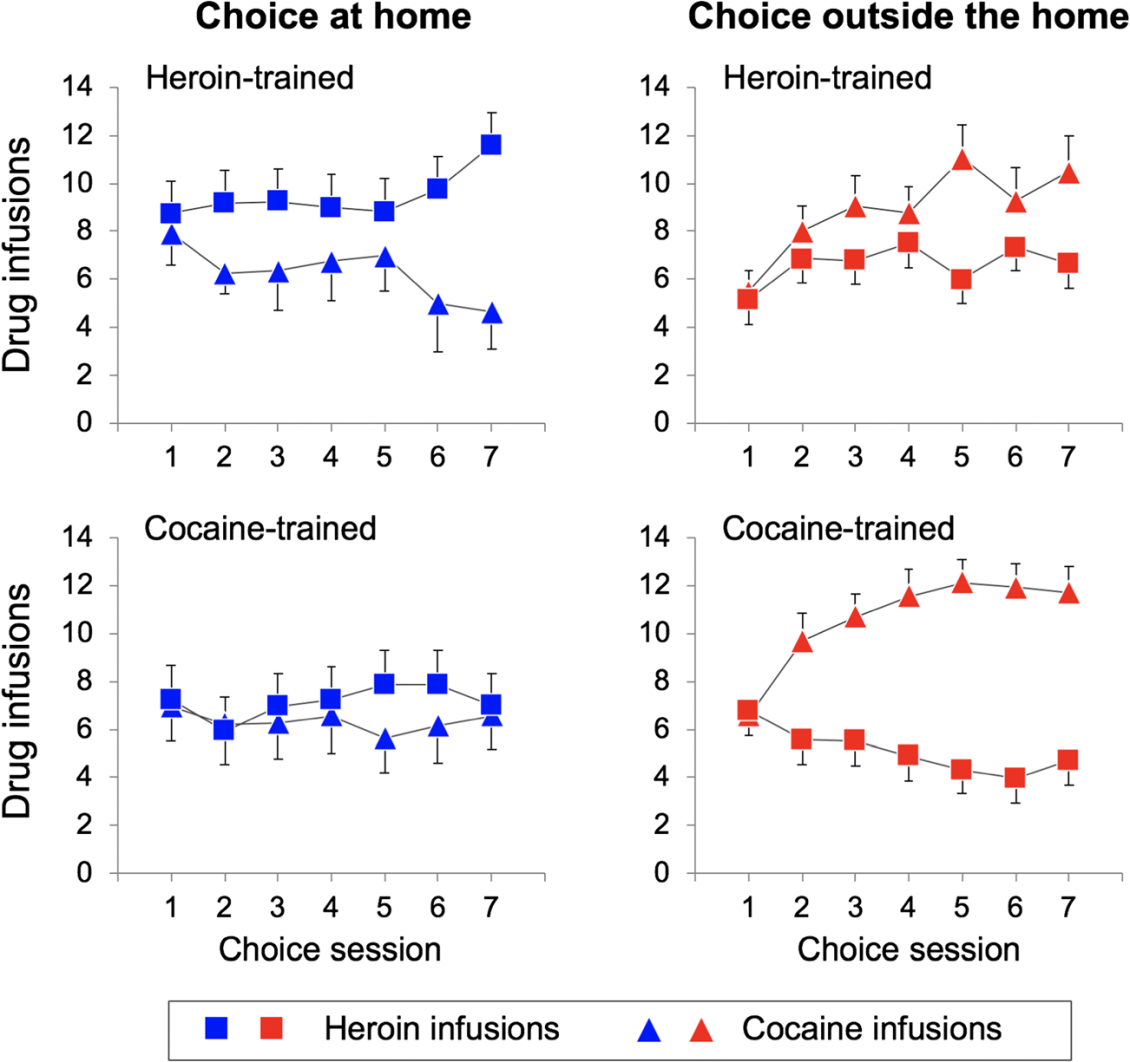
Mean (±SEM) number of cocaine versus heroin infusions, during the seven sessions of the choice phase, as a function of setting and drug history. See text for details about statistical analysis

**Fig. 4:**
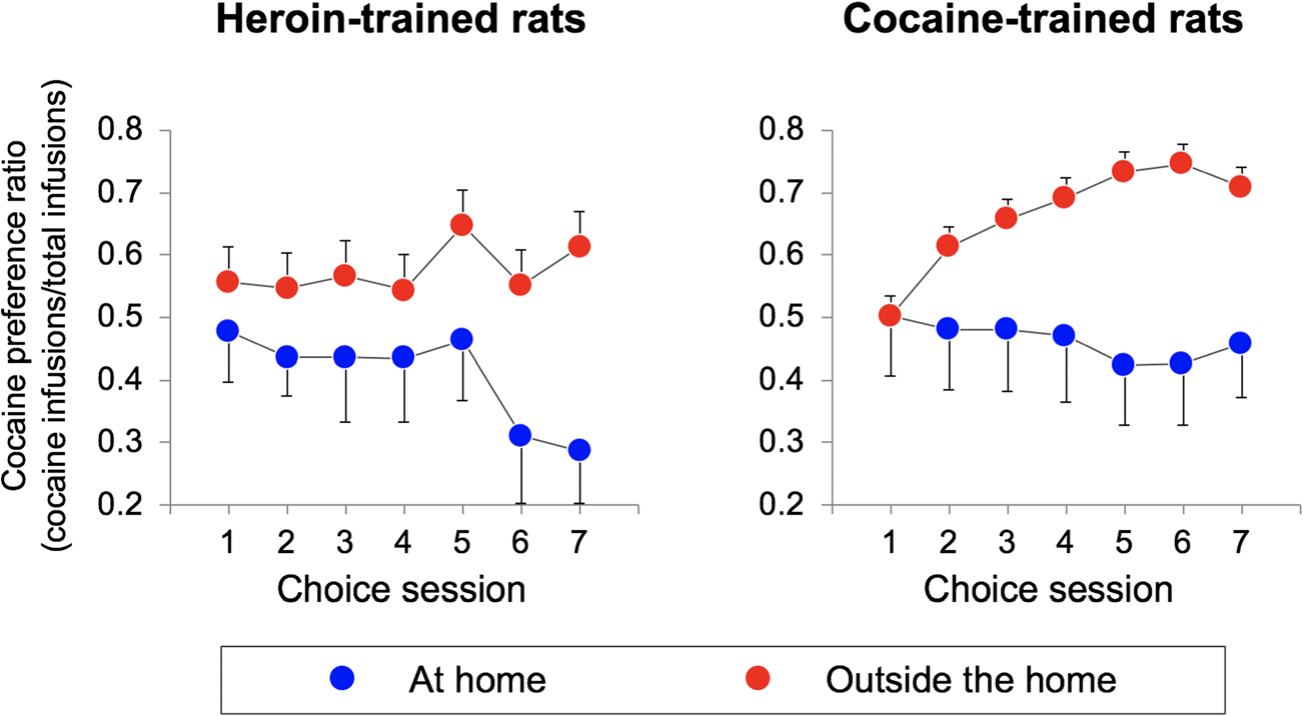
Mean (±SEM) cocaine preference ratio (the ratio between the number of cocaine infusion and the total number of infusions), as a function of setting and drug history. See text for details about statistical analysis

**Fig. 5:**
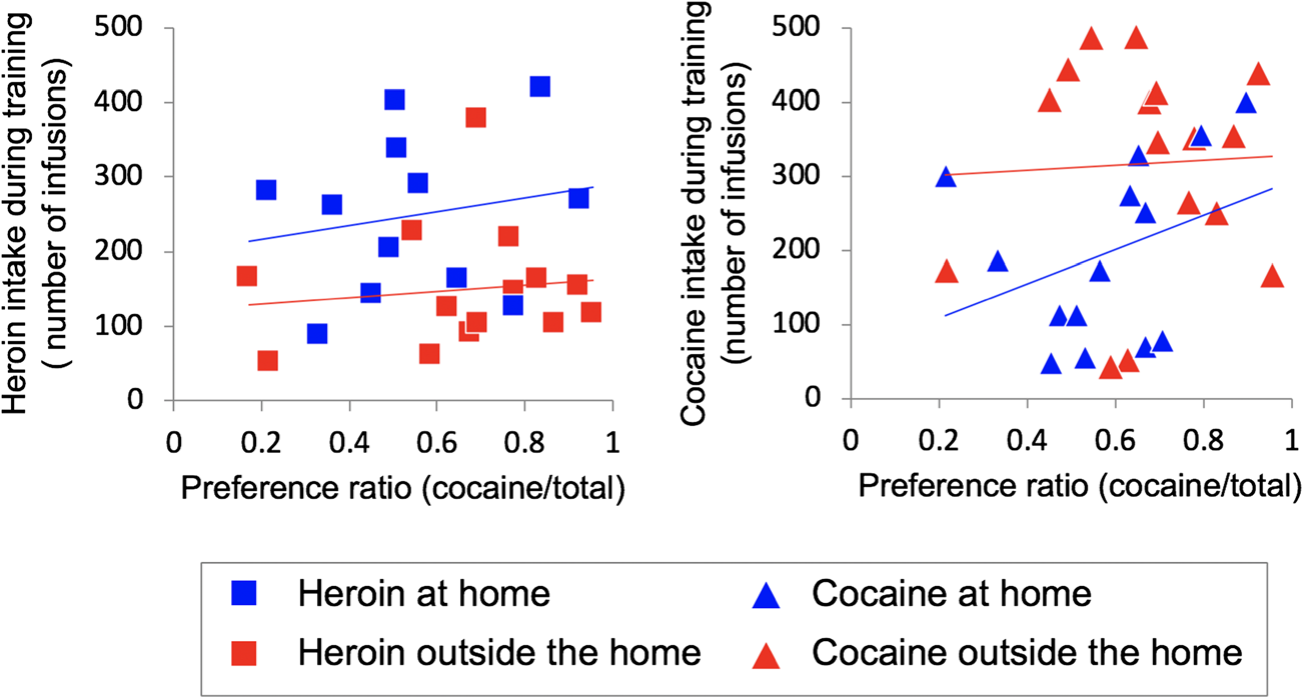
Linear correlation analysis between preference ratios and total amounts of heroin or cocaine taken during the training phase, as function of setting. See text for details about statistical analysis

Figure 3 illustrates the number of cocaine versus heroin infusions, during the seven sessions of the choice phase, as a function of the setting. The rats tested outside the home made a slightly higher number of total choices relative to the rats tested at home (109.2 ± 2.1 vs. 101.7 ± 4.4) but this difference was not significant (*p* = 0.13). As detailed in the “Data analysis and statistics” section, the comparison of cocaine versus heroin infusions would be meaningless because the two variables were inversely related. However, Fig. 4 illustrates the cocaine preference ratio (the ratio between the number of cocaine infusion and the total number of infusions) calculated on the same data. During the first choice session, the rats self-administered more or less the same amounts of heroin and cocaine infusions, indicating that no lever bias had developed during training. However, on the following sessions, preferences emerged. The rats tested outside the home exhibited higher cocaine preference ratio than the rats tested at home, as indicated by a significant main effect of *setting* (*F*_1, 52_ = 5.718; *p* = 0.02, *η*^2^ = 0.099). These differences grew larger over test session, even though the interaction between *choice session* and *setting* only approached significance (*F*_6, 312_ = 2.078; *p* = 0.056, *η*^2^ = 0.038). In contrast, drug history appeared to exert only a modest influence on drug choice, as indicated by the lack of a main effect of *drug history* (*F*_1, 52_ = 1.835, *p* = 0.18, *η*^2^ = 0.034), with no *setting* × *drug history* (*F*_1, 52_ = 0.050, *p* = 0.825, *η*^2^ = 0.001), *choice session* × *drug history* (*F*_6, 312_ = 0.622, *p* = 0.713, *η*^2^ = 0.012), or *choice session* × *setting* × *drug history* (*F*_6, 312_ = 0.606, *p* = 0.725, *η*^2^ = 0.012) interaction. Finally, as shown in Fig. 5, there was no significant correlation between preference ratios and total amounts of heroin or cocaine taken during the training phase either at home (*r*^2^ = 0.03, *p* = 0.47 for heroin; *r*^2^ = 0.12, *p* = 0.23 for cocaine) or outside the home (*r*^2^ = 0.03, *p* = 0.20 for heroin; *r*^2^ = 0.002, *p* = 0.87 for cocaine).

**Fig. 6:**
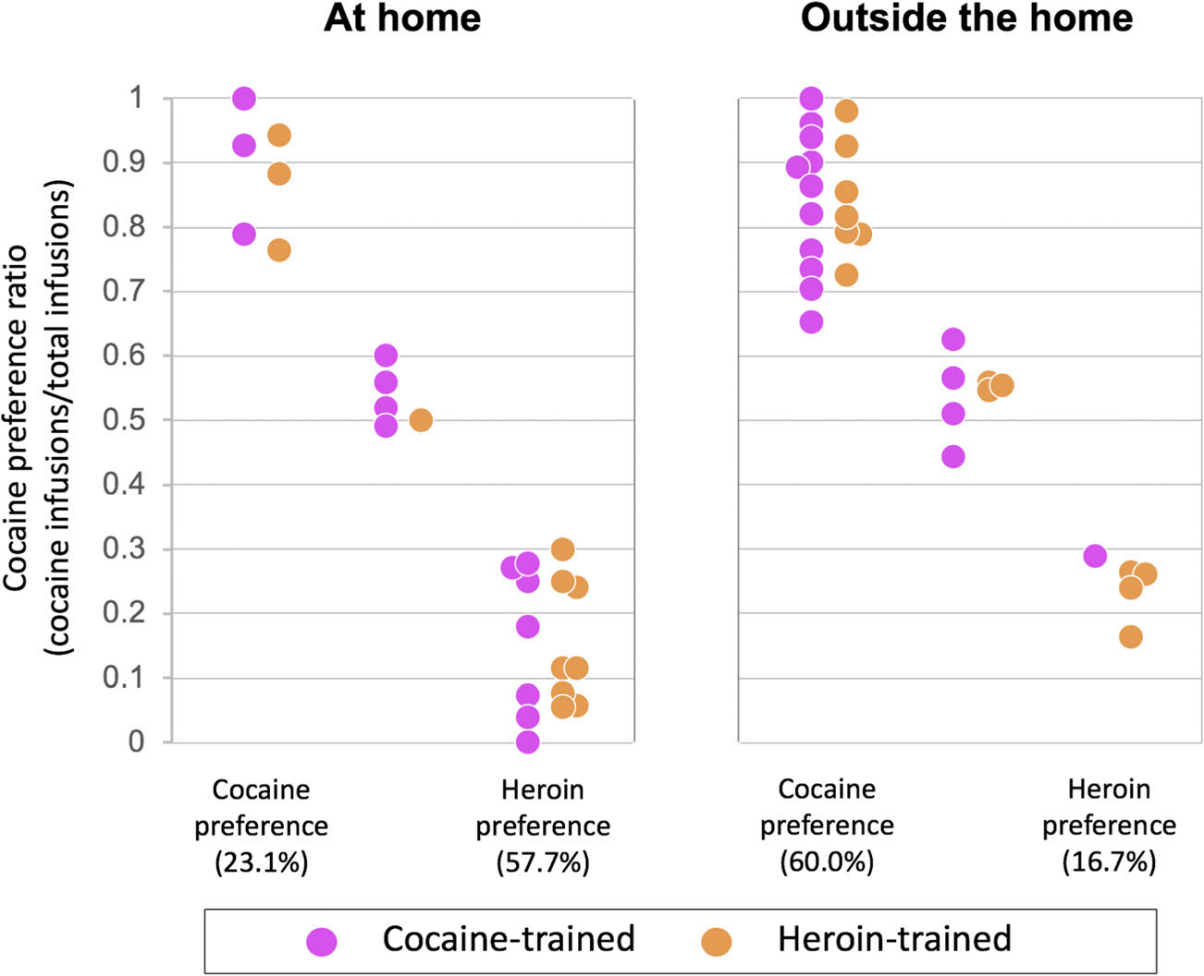
Drug preferences in individual rats (calculated using bootstrapping analysis), as a function of setting and drug history. See text for details about statistical analysis

Figure 6 illustrates drug preferences in individual rats. Most rats (80.4%) expressed a significant preference for either heroin or cocaine (see “Data analysis and statistics”). Individual preferences were influenced by the setting: outside the home, there were many more cocaine-preferring rats (60.0%) than heroin-preferring rats (16.7%), whereas the at home, there were many more heroin-preferring rats (57.7%) than cocaine-preferring rats (23.1%), with a near tenfold shift in cocaine versus heroin preference as a function of setting.

## Discussion

An unbiased choice procedure was used here to determine drug preferences in rats previously trained to self-administer heroin or cocaine in different settings. We found that individual differences in drug preference were powerfully influenced by the setting of drug taking but in a substance-specific manner. At home, two and a half times more rats chose heroin than cocaine. The opposite occurred outside the home: four times more rats chose cocaine than heroin. Overall, there was a nearly tenfold shift in drug preferences as a function of setting. Most important, we found that drug history had a negligible effect on drug preference, that is, at home the rats preferred heroin even if they had previously self-administered only cocaine whereas outside the home the rats preferred cocaine even if they had previously self-administered only heroin.

### Individual differences in heroin versus cocaine preference

Research done in the last three decades has stressed the existence of shared neural substrates for the rewarding effects of addictive drugs (Di Chiara and Imperato 1988; Nestler 2004; Badiani et al. 2011; Covey et al. 2014). Psychostimulants and opiates, for example, despite their very different pharmacodynamic profiles, share the ability to increase dopamine levels in the terminal regions of the mesostriatal dopamine system. Cocaine and amphetamine do so by binding the dopamine transporter located on dopaminergic terminals (Harris and Baldessarini 1973; Rothman and Baumann 2003). Opiates act indirectly by activating mu opioid receptors (Inturrisi et al. 1983; Selley et al. 2001) located on GABAergic interneurons, which exert tonic inhibitory control on dopaminergic neurons in the ventral tegmental area (Gysling and Wang 1983; Di Chiara and Imperato 1986; Johnson and North 1992). The evidence of shared mechanisms of action has led to the widespread assumption that the rewarding effects of psychostimulants and opiates are substantially the same. It is also thought that psychostimulants and opiates induce partly overlapping neuroplastic changes that are responsible for the transition to abuse and the vulnerability to relapse (Robinson and Berridge 1993; Berridge and Robinson 2016; Nestler 2001, 2004; Pickens et al. 2011). However, unified models of drug reward cannot easily provide a satisfactory explanation for the existence of drug preferences of the type reported here. In particular, it would be difficult to explain why some of the rats that had previously self-administered only cocaine should then prefer heroin, and, vice versa, some of the rats that had previously self-administered only heroin should then prefer cocaine. It is reasonable to conclude that heroin and cocaine produce distinct internal states and that the rewarding values of these internal states differ substantially from one individual to another.

Further research is necessary to determine the neurobiological correlates of cocaine versus heroin reward in rats. However, it is of some interest that within-subject single-unit electrophysiology experiments in rats have shown that heroin and cocaine self-administration engage distinct neuronal populations in the terminal regions of the mesostriatal dopamine system (Chang et al. 1998). Moreover, studies using chemical lesions (Pettit et al. 1984; Gerrits and Van Ree 1996), dopamine receptor antagonists (Ettenberg et al. 1982), and RNA-interference of dopamine D1 receptors (Pisanu et al. 2015) have shown differential involvement of the dopaminergic system in cocaine versus heroin self-administration.

### Heroin versus cocaine preference as a function of setting

The findings reported here indicate that drug preferences are a function not only of individual differences but also of the setting of drug use, confirming a previous report (Caprioli et al. 2009).

We have previously shown that the setting can influence in opposite directions virtually all aspects of cocaine versus heroin reward, including drug intake, motivation to work for the drug, drug discrimination, drug affect, and vulnerability to relapse into heroin or cocaine seeking after a period of abstinence (Paolone et al. 2004; Caprioli et al. 2007a, b, 2008; Celentano et al. 2009; Montanari et al. 2015; Avvisati et al. 2016). In contrast, earlier studies focusing on drug-induced psychomotor sensitization have shown that the magnitude of sensitization to *both* psychostimulants and opiates is much greater when these drugs are repeatedly administered outside the home than when administered at home (Badiani et al. 1995, a, b, 1997, 2000; Browman et al. 1998a, b; Crombag et al. 1996, 2000; Fraioli et al. 1999; Ostrander et al. 2003; Paolone et al. 2003, 2007). This suggests a fundamental dissociation between the rewarding effects of opiates and their psychomotor-activating effects and cautions against the long-standing notion that the latter necessarily reflect the former (Wise and Bozarth 1987). We also found that the analgesic response to morphine and development of tolerance to this effect is the same at home and outside the home (Paolone et al. 2003), consistent with the notion that tolerance and sensitization to different drug effects can develop independent of each other (Stewart and Badiani 1993). Taken together, these findings indicate that the influence of setting on drug reward is not the consequence of a general facilitation or reduction in drug efficacy due to changes in pharmacokinetics or pharmacodynamics.

As discussed in the “Introduction,” to account for the ability of the setting to influence in opposite ways the reinforcing effects of heroin and cocaine, we have proposed that the overall rewarding effects of addictive drugs are the result of a complex interaction between their central and peripheral effects and the setting of drug use (Badiani 2013). In the presence of mismatch between exteroceptive information (setting) and interoceptive information generated by central and peripheral drug actions, the affective valence of drug experience would be more negative than in conditions in which there was no such a mismatch. Self-administration experiments with other classes of drugs with sedative or activating effects lend support to our hypothesis (Testa et al. 2011; De Luca and Badiani 2011; De Luca et al. 2012).

The major aim of the present study was to test a crucial implication of the mismatch theory, that is, that rats would tend to prefer heroin to cocaine at home, and cocaine to heroin outside the home, regardless of whether they had previously self-administered only heroin or only cocaine. Overall, our findings are consistent with this hypothesis. Yet, the extent to which drug choice was independent of drug history was somewhat surprising, as it suggests that, under certain conditions, associative learning processes (Childress et al. 1993; Grimm et al. 2001) and drug-induced neuroplastic adaptations (for reviews, see Robinson and Berridge 1993; Nestler 2001; Pickens et al. 2011) play a relatively minor role in shaping the preference of an individual for one drug or the other.

The neurobiological mechanisms through which the setting influences drug preferences in the rat are not known. However, it has been shown that the setting modulates in different, sometimes opposite manner the activity of reward areas of the brain in response to psychostimulants, such as amphetamine and cocaine (Badiani et al. 1998, 1999; Uslaner et al. 2001a, b; Ostrander et al. 2003; Hope et al. 2006), versus opiates, such as morphine and heroin (Ferguson et al. 2004; Paolone et al. 2007; Celentano et al. 2009). In particular, it appears that cocaine increases (relative to vehicle) the activity of D2+/enkephalin+ medium spiny neurons (MSN) of the striatum, to a much greater extent outside the home than at home (Uslaner et al. 2001b), whereas the opposite occurs with morphine (Ferguson et al. 2004). These earlier findings might have some bearing to the results reported here given that D2+/enkephalin+ MSN indirectly disinhibit the subthalamic nucleus (STN), which has been implicated in reward and decision-making (Zenon et al. 2016; Pelloux et al. 2018). Further work is necessary to explore the role of the STN in drug preferences.

## Conclusions

The findings reported here have important implications for addressing a crucial issue in drug addiction research. That is, to what extent “the risk factors for the use or misuse of a particular class of psychoactive substances are specific to that class or are nonspecific in that they predispose the individual to the use or misuse of a wide range of such compounds” (Kendler et al. 2003, p. 687). Studies in twins (Tsuang et al. 1998; Kendler et al. 2003) have shown that the substance specificity of drug abuse is almost entirely due to environmental influences but very little is known about their nature (Zinberg 1984). Thus, it is remarkable that translational studies in humans have shown that the setting can influence drug reward in a manner similar to that observed in the rat. Indeed, polydrug users with a diagnosis of substance use disorder (SUD) reported a preference for using heroin at home and cocaine outside the home (Caprioli et al. 2009; Badiani and Spagnolo 2013). This was true regardless of the route of administration or of social context. A recent study has shown that such preferences may depend on the fact that heroin and cocaine produce different affective states in different settings (De Pirro et al. 2018). In particular, it was found that in cocaine and heroin users with SUD, the affective valence of heroin was greater at home than outside the home, whereas the opposite was seen for cocaine. Furthermore, in these individuals, the setting exerted a substance-specific influence also on the activity of brain regions implicated in processing drug reward and contextual information (De Pirro et al. 2018). Taken together, our work suggests that unitary constructs of drug reward and drug addiction should be revised in the light of mounting evidence indicating distinct neurobiological underpinnings for the response to different classes of drugs (see Badiani et al. 2011; Badiani 2013; Badiani et al. 2018; Peters et al. 2013; Nutt et al. 2015).
